# Anatomical classification of accessory foramina in human mandibles of adults, infants, and fetuses

**DOI:** 10.1007/s12565-012-0136-z

**Published:** 2012-05-03

**Authors:** Agnieszka Przystańska, Małgorzata Bruska

**Affiliations:** Department of Anatomy, Poznan University of Medical Sciences, 6 Święcicki Street, 60-781 Poznan, Poland

**Keywords:** Mandible, Accessory foramina, Neurovascular bundle, Genial tubercle

## Abstract

In the past few decades, a number of studies have reported that accessory foramina are located on the internal aspect of the mandible, indicating their potential importance for effective and successful clinical procedures. The aim of this study is to evaluate adult, infantile, and fetal human mandibles for occurrence of accessory foramina. To our knowledge, this is the first time that an attempt has been made to examine their particular co-location using a systematic approach and perspective. A total of 397 human mandibles, including 299 adult, 18 infantile, and 80 fetal, were investigated macroscopically for the frequency, position, and diameter of accessory foramina. In 96 % of investigated adult mandibles, at least one accessory foramen was found. Foramina were located either superior or inferior to the genial tubercle, as well as lateral to the tubercle. Bearing in mind their usual location, four different types of coexistence of foramina were distinguished. Accessory foramina were also present in similar locations in infantile and fetal mandibles. Accessory mandibular foramina are constant structures of human mandible. Their frequency, size, and location vary depending on the type of the foramen. Observations on children and fetal mandibles showed no significant differences in evaluation of accessory foramina, with the exception of lower occurrence in this group of subjects.

## Introduction

In the mandible, accessory foramina refer to all mandible openings excluding the mandibular and mental foramina, and the alveolar sockets (Sutton 1974). Numerous reports have confirmed the presence of accessory foramina in the human mandible, but their classification and nomenclature are not identical (Chapnick 1980; Fanibunda and Matthews 1999, 2000; Haveman and Tebo 1976; Jeyaseelan and Sharma 1984; Katakami et al. 2009; Kaufman et al. 2000; Kawai et al. 2009; Liang et al. 2004, 2006, 2007; McDonnell et al. 1994; Ossenberg 1987; Pyle et al. 1999; Rosano et al. 2009; Shiller and Wiswell 1954; Shirai 1960; Sutton 1974; Yoshida et al. 2005; Zivanovic 1970).

It was reported that accessory foramina are present more often on the internal surface of the mandible (Chapnick 1980; Fanibunda and Matthews 1999, 2000; Haveman and Tebo 1976; Sutton 1974) than on its external one. They are located more frequently in the anterior part of the mandible (Fanibunda and Matthews 1999; Sutton 1974), though they have also been observed in the retromolar area (Haveman and Tebo 1976; McDonnell et al. 1994; Ossenberg 1987; Zivanovic 1970; Carter and Keen 1971) or the ramus of the mandible (Casey 1978; Barker and Locket 1972).

A vast majority of researchers found the accessory foramen lying in the midline, either superior or within the genial tubercle, and named it therefore the lingual foramen (McDonnell et al. 1994; Shiller and Wiswell 1954). Sutton (1974) referred to this as the midline foramen, Madeira et al. (1978) as the superior retromental foramen, whereas Eriguchi (1954) and Shirai (1960) as the supraspinous foramen.

There are also different names used for accessory foramina lying in the midline, inferior to the genial tubercle; foramina in this location are referred to as the inferior lingual foramen (Shiller and Wiswell 1954), the inferior retromental foramen (Madeira et al. 1978), or the infraspinous foramen (Shiller and Wiswell 1954; Eriguchi 1954).

Accessory foramina located in the lateral regions of the mandible are described as lateral retromental foramina (Madeira et al. 1978) or internal mental foramina (Jeyaseelan and Sharma 1984).

The undefined topography of the accessory mandibular foramina created some challenges for us, and our observations result in the first presentation of the types of coexistence of accessory foramina.

The current literature lacks observational evidence on accessory foramina in human mandibles of different age groups. This therefore inspired us to conduct macroscopic observations, and to determine whether accessory foramina are constant structures in infantile and fetal mandibles, and if their location and topography differ from those observed in adults.

## Materials and methods

The study was undertaken on 397 dry mandibles, including 299 adult (of undetermined sex) mandibles, 18 mandibles of children, and 80 fetal (from 18 to 38 weeks) mandibles. The bones were taken from a collection of the Department of Anatomy, Poznań University of Medical Sciences in Poznań, and from a collection of the Institute of Anthropology, Adam Mickiewicz University of Poznań.

The topography and frequency of accessory foramina on the internal surface of the mandibular body were investigated macroscopically. The diameter of the foramina was established using scaled wires (Ø 0.06–1.2 mm). To establish the exact location of the foramina, the height of the mandibular body was measured from the lowermost point of the symphysis lying in the midline to the uppermost point of the alveolar part of the mandible, lying between the alveolar juga of the central incisors.

The distance between the inferior border of the mandible and the foramen, and their location with respect to the genial tubercle, were established. The horizontal location of the foramen in relation to the mandibular body height was expressed in %. In case of lateral foramina, the distance from the midline to the foramen, as well as the distance from the inferior border of the mandible to the foramen, were measured to establish the position.

## Results

### Adult mandibles

The average height of the mandibular bodies was 29 mm (from 16 to 42 mm). The total number of accessory foramina found on the internal surface of the bodies in all adult mandibles was 700. The number of foramina ranged from 0 (in 12 mandibles) to 7 foramina (in 2 mandibles). In 288 specimens (96 %), at least one accessory foramen was present on the internal surface of the mandibular body.

**Fig. 1:**
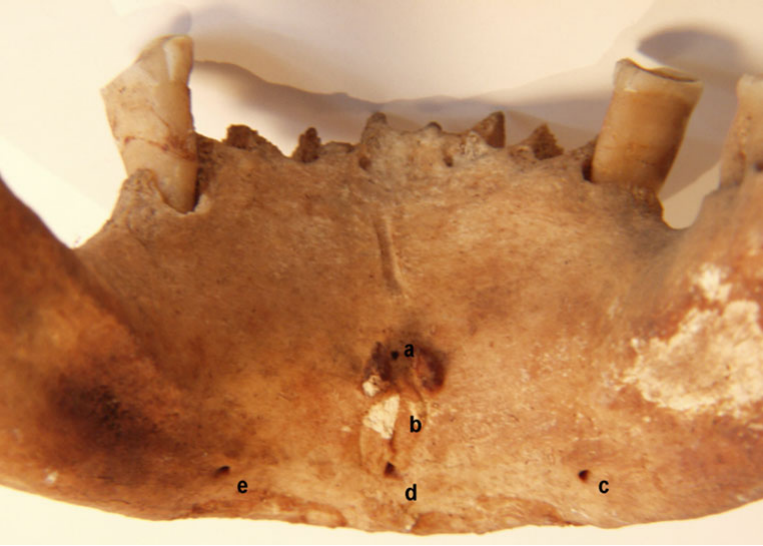
Accessory foramina of human mandibular body. *a* Foramen superior to genial tubercle, *b* genial tubercle, *c*, *e* foramina lateral to genial tubercle, *d* foramen inferior to genial tubercle

There were four different types of foramina found on the internal surface of the mandibular bodies:

- Accessory foramen within or superior to the genial tubercle
- Accessory foramen inferior to the genial tubercle
- Accessory foramina lateral to the genial tubercle (Fig. 1)

.

In 276 mandibles (92 %), at least one accessory foramen located in the midline was observed (either superior or inferior to the genial tubercle).

**Fig. 2:**
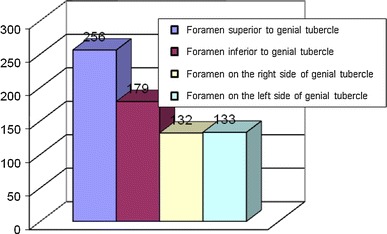
Incidence of accessory foramina on the internal surface of the human adult mandibular body

**Fig. 3:**
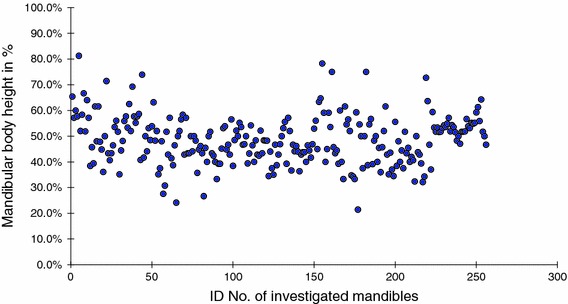
Position of the accessory foramen in adults superior to the genial tubercle in relation to the total height of the mandibular body

Accessory foramen located in the midline and superior to the genial tubercle was observed in 256 (86 %) mandibles (Fig. 2). The average position of the foramen was 48 % of the mandibular body height. Its position ranged from 21 to 81 % of the total height of the mandibular body (Fig. 3), and its diameter ranged from 0.3 to 1.5 mm.

**Fig. 4:**
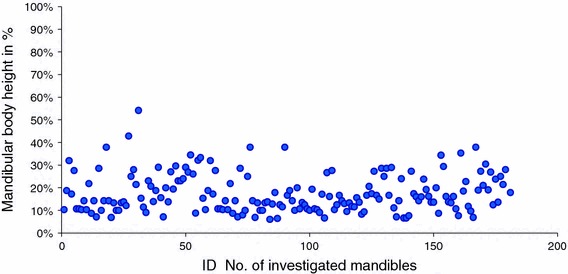
Position of the accessory foramen in adults inferior to the genial tubercle in relation to the total height of the mandibular body

Accessory foramen inferior to the genial tubercle was observed in 179 (60 %) mandibles (Fig. 2). It was located between 6 and 54 % of the total mandibular body height (Fig. 4). In 166 (93 %) mandibles, it was found in the inferior third of the mandibular body. In 12 (7 %) specimens, the foramen was twofold. Its diameter varied from 0.1 to 1.5 mm.

Accessory foramina lateral to the genial tubercle were located uni- or bilaterally. There were never more than two lateral foramina in one mandible. In 133 mandibles, the foramen was observed on the left side, and on the right in 132 mandibles (Fig. 2). In 109 mandibles, it was found bilaterally. The average position of the foramina was observed as 26 % of the mandibular body height, and in almost 90 % of the investigated mandibles, they were located in the lower third of mandible. The average distance between the foramen and the midline was 15.4 mm (left) and 15.2 mm (right). The diameter of the foramen varied from 0.1 to 1.5 mm.

In reference to the recurrent topographical patterns of the accessory foramina, an attempt was made to distinguish the particular types of their coexistence (Table 1). The accessory foramina coexisted in four different types:

**Table 1:** Incidence of accessory foramina in the mandibles of adults, children, and fetuses

	Adult	Children	Fetuses
Total number of investigated mandibles	299	18	80
Total number of foramina found	700	33	72
Accessory foramen superior to genial tubercle	256 (86 %)	14 (78 %)	25 (31 %)
Accessory foramen inferior to genial tubercle	179 (60 %)	9 (50 %)	9 (11 %)
Accessory foramen lateral to genial tubercle (right)	132 (44 %)	5 (28 %)	12 (15 %)
Accessory foramen lateral to genial tubercle (left)	133 (44 %)	5 (28 %)	16 (20 %)
Type I	130 (43 %) including: type Ia-66 Ib-52 Ic-12	9 including: type Ia-6 Ib-3 Ic-0	27 (34 %) including: type Ia-3 Ib-14 Ic-10
Type II	146 (49 %) including: type IIa-93 IIb-45 IIc-8	5 (28 %) including: type IIa-3 IIb- 2 IIc-0	12 (15 %) including: type IIa-1 IIb-7 IIc-4
Type III	10 (3 %)	0 (0 %)	10 (12 %)
Type IV	13 (4 %)	4 (22 %)	31 (39 %)

**Fig. 5:**
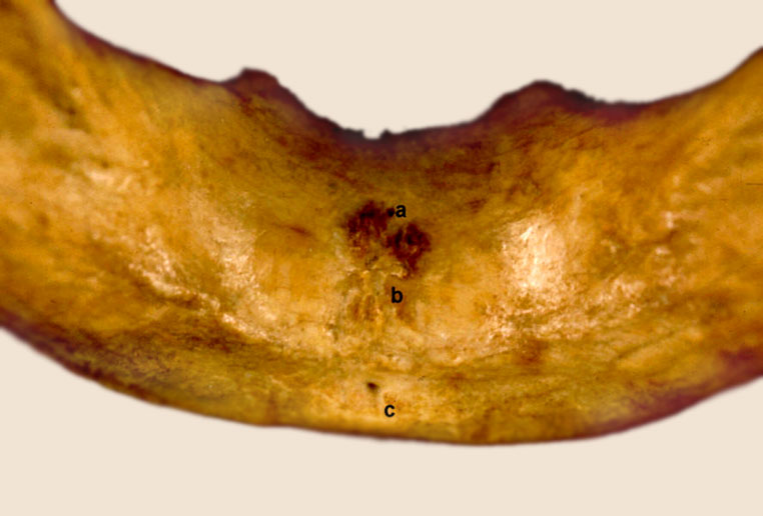
Type I. *a* Foramen superior to genial tubercle, *b* genial tubercle, *c* foramen inferior to genial tubercle
- Type Ib: one single foramen located in the midline, superior to or within the genial tubercle (52 cases)
- Type Ic: one single foramen located inferior to the genial tubercle, observed in 12 (4 %) of the mandibles.

Type I was present in 130 mandibles. It was subdivided into 3 groups:

- Type Ia: both midline foramina, superior and inferior to the genial tubercle, being observed in 66 (22 %) cases (Fig. 5)

**Fig. 6:**
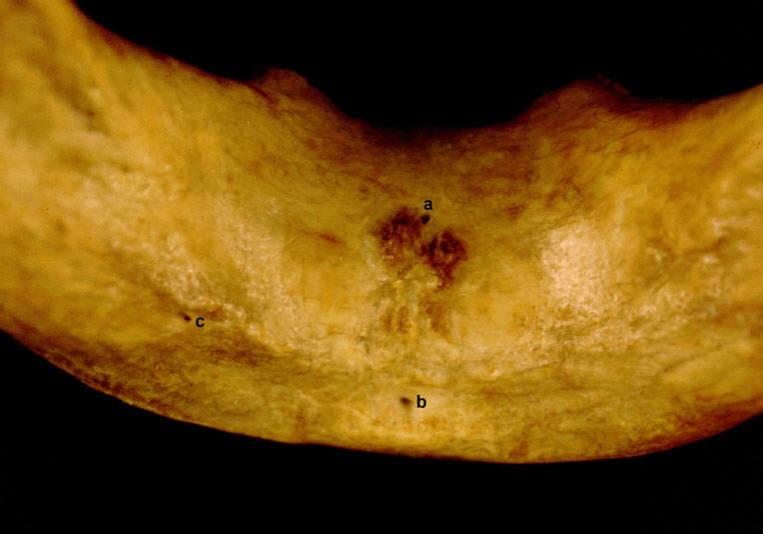
Type IIa. *a* Midline foramen superior to genial tubercle, *b* midline foramen inferior to genial tubercle, *c* foramen lateral to genial tubercle
- Type IIb, observed in 45 cases (15 %): midline foramen located superior to the genial tubercle, accompanied by either one or both lateral foramina
- Type IIc, observed in 8 cases (3 %): midline foramen located inferiorly to the genial tubercle, accompanied by one or two lateral foramina.

Type II was characterized by the presence of accessory midline foramina accompanied by lateral foramina. It was observed in 146 cases. It was subdivided into 3 groups:

- Type IIa was observed in 93 (31 %) mandibles. Two midline foramina were present (superior and inferior to the genial tubercle), and they were accompanied by either one or two lateral foramina (Fig. 6).

**Fig. 7:**
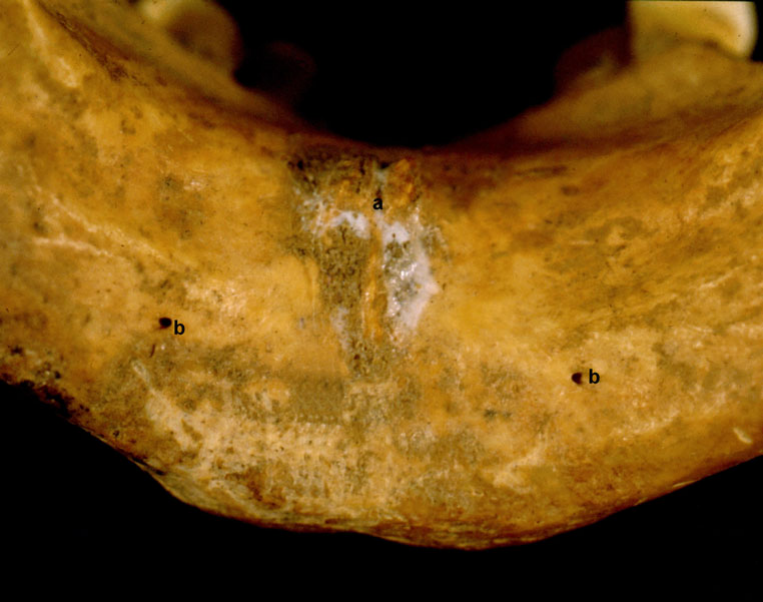
Type III. *a* Genial tubercle, *b* foramina lateral to genial tubercle

In type III, only foramina lateral to the genial tubercle were observed. It was found in 10 mandibles (3.4 %). In 5 specimens, a single, unilateral foramen was present, whereas in 5 mandibles, bilateral foramina were found (Fig. 7).

Type IV included 13 mandibles with no foramina.

### Mandibles of children

**Fig. 8:**
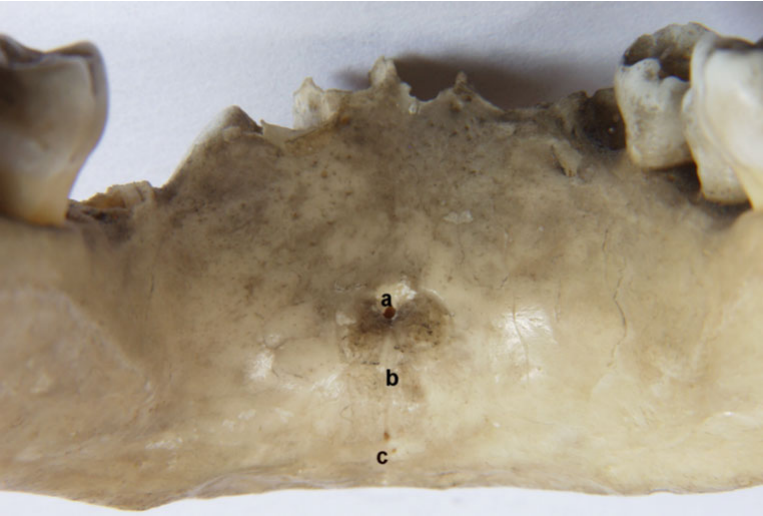
Accessory foramina in child. *a* Foramen superior to genial tubercle, *b* genial tubercle, *c* double foramen inferior to genial tubercle

In the mandibles of 18 children that we examined, a total of 33 accessory foramina were found (Fig. 8), and their number in mandibles varied form 0 (4 cases) to 4 (3 cases).

**Fig. 9:**
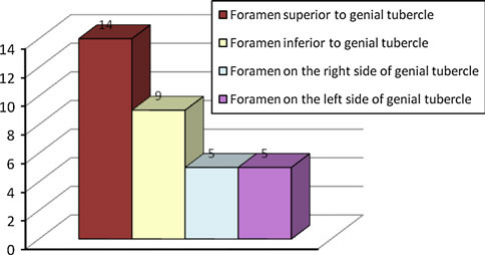
Incidence of accessory foramina on the internal surface body in mandibles of children

In 14 mandibles (78 %), at least one accessory foramen was observed, and in all cases, it was located in the midline, just above the genial tubercle (Fig. 9).

Accessory foramen lying inferior to the genial tubercle was observed in 9 mandibles (50 %). In two cases, it was twofold. In 5 mandibles (28 %), foramina lying laterally to the genial tubercle were found, and they were bilateral in all cases. Type Ia was observed six times, and therefore with the highest frequency; type Ib and IIa were observed three times each, and type IIb was found twice. Types Ic, IIc, and III were absent. Type IV was found in 4 mandibles.

### Fetal mandibles

**Fig. 10:**
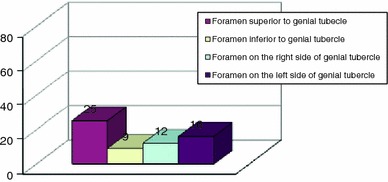
Incidence of accessory foramina on the internal surface of the mandibular body in human fetuses

Of the 80 fetal mandibles investigated—from 4 to 9 months intrauterine period—a total of 72 accessory foramina were found on the internal surface of the mandible (Fig. 10). The number of foramina ranged from 0 (in 31 mandibles) up to 3 (in 5 cases). In 49 mandibles (61 %), at least one accessory foramen on the internal surface of the mandibular body was found. In 39 cases (49 %), a single foramen lying in the midline was observed. However, a foramen superior to the genial tubercle was present in 25 cases (31 %), and inferior to the genial tubercle in 19 cases (24 %). In 5 mandibles (6 %), both foramina were observed. Foramen lying lateral to the genial tubercle was observed in 12 cases (15 %) on the right, and on the left in 16 cases (20 %) (Fig. 10). In 6 mandibles (7 %), they were bilateral.

The diameter of the foramina in fetal mandibles ranged from 0.06 to 0.3 mm.

The accessory foramina in fetal mandibles formed type Ib in 14 cases, and type Ic and type III in 10 cases each. Type IIb was observed in 7, type IIc in 4, and type Ia in 3 mandibles. Type IIa was found only once. Type IV was found in 31 mandibles.

**Fig. 11:**
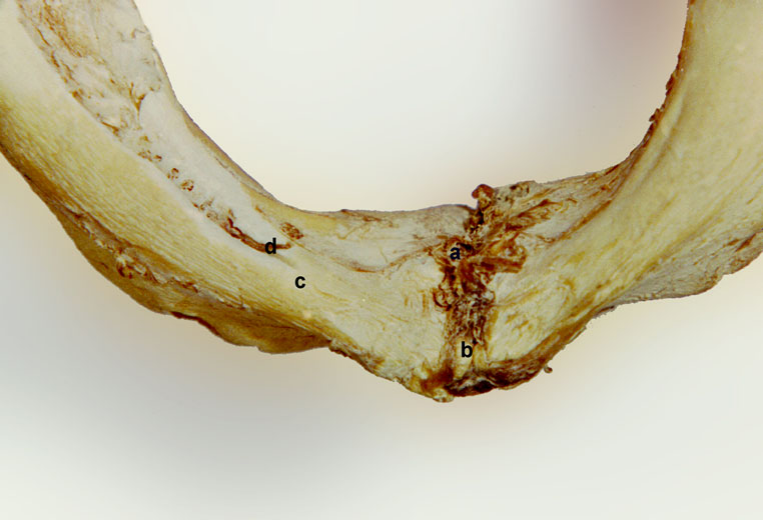
Fetal mandible. *a* Accessory foramen superior to genial tubercle, *b* accessory foramen inferior to genial tubercle, *c* accessory foramen lateral to genial tubercle, *d* structure entering the foramen

In some fetal mandibles, it was observed that the accessory foramina opened into the short interosseous canal that was directed anterolaterally and joined the mandibular canal (Fig. 11).

## Discussion

The presence of accessory foramina has been reported in many descriptive reports. Numerous studies which examined foramina have confirmed their presence and, furthermore, discussed their potential role in neurovascular transmission and possible clinical significance (Longoni et al. 2007; Gahleitner et al. 2001; Jacobs et al. 2007; Nagar et al. 2001; Przystańska and Bruska 2010; Tagaya et al. 2009). Morphological investigations of accessory foramina did not determine their detailed topography. Based on the literature reports, the frequency of accessory foramina differs, depending on the number of investigating mandibles and on the methods and criteria applied.

A vast majority of the morphological reports confirmed that accessory foramina occur in nearly all investigated mandibles (Shiller and Wiswell 1954; Sutton 1974). In contrast, it has been reported (Chapnick 1980) that the frequency of appearance of accessory foramina in mandible is much lower (68.9 %), although this study was performed on only 122 mandibles and only foramina larger than 0.5 mm in diameter were considered. Attempts to determine the topography of accessory foramina by using modern cone-beam computed tomography (Katakami et al. 2009; Kawai et al. 2009; Liang et al. 2006; Yoshida et al. 2005) reported evidence of accessory foramina in up to 100 % of investigated mandibles.

It is reported that, in the majority of investigated mandibles, at least one accessory foramen located on the internal surface is present (Baldissera and Silveira 2002; Butch 1978; Fanibunda and Matthews 1999; Katakami et al. 2009; Kaufman et al. 2000; Kawai et al. 2009; Liang et al. 2006, 2007; Shirai 1960; Sutton 1974). Different criteria for foramina qualification, and their acknowledgement within the study, may lead to overall underestimation of foramina. In the investigation of 122 human mandibles reported by Chapnick (1980), the incidence rate of foramina was 68.9 %; however, foramina with diameter of 0.5 mm and higher were included in that study.

Our results confirm the observations made (Baldissera and Silveira 2002; Eriguchi 1954; Fanibunda and Matthews 1999; Kawai et al. 2009; Sutton 1974) indicating that a vast majority of foramina are located in the symphyseal region of the mandible, being observed in most mandibles (Baldissera and Silveira 2002; Eriguchi 1954; Fanibunda and Matthews 1999; Kaufman et al. 2000; Shiller and Wiswell 1954; Sutton 1974).

Taking all the accessory foramina into consideration, it seems that foramina located in the midline appear with the highest incidence rate. Accessory foramen located superior to the genial tubercle is the most frequent (Eriguchi 1954; Shiller and Wiswell 1954; Sutton 1974); however, the rate of its appearance reported in literature differs (Sutton 1974; Fanibunda and Matthews 1999; Liang et al. 2004; Pyle et al. 1999; Rosano et al. 2009; Shiller and Wiswell 1954; Eriguchi 1954), reaching 99 % (Liang et al. 2004) and 100 % (Baldissera and Silveira 2002). Only some investigators mentioned its dual appearance (Shiller and Wiswell 1954). The diameter of this foramen ranged from 0.1 mm (Shiller and Wiswell 1954) to more than 1.5 mm (Shirai 1960). The frequency of the second midline foramen, located inferior to the genial tubercle, is lower (Shiller and Wiswell 1954; Eriguchi 1954), reaching an average of 60 % of mandibles.

On the internal surface of the mandible, accessory foramina located lateral to the genial tubercle are observed with frequency ranging from 30 % (Fanibunda and Matthews 1999; Carter and Keen 1971), 60 % (Haveman and Tebo 1976; Shiller and Wiswell 1954), 70 % (Fanibunda and Matthews 1999; Sutton 1974), up to 80 % (Eriguchi 1954). The different methods applied in determining their topography create confusion. It was suggested that their location depends on the attachments of muscles (Carter and Keen 1971; Eriguchi 1954; Jeyaseelan and Sharma 1984; Sutton 1974), and that they are bilateral structures (Fanibunda and Matthews 1999; Haveman and Tebo 1976; Kaufman et al. 2000). Our observations confirm their bilateral occurrence in 36 % of cases.

Current literature shows no evidence of attempts to distinguish the different types of coexistence of accessory foramina. We observed that they form repeated patterns, which we referred to as “types” according to their location on the internal surface of the mandible.

According to studies of the mandibular body, accessory foramina located in the alveolar part, between the alveolar sockets in the lower incisors region, were also distinguished. Detailed information, including the frequency, size, and topography of these foramina, was previously reported (Przystańska and Bruska 2005).

The idea of conducting studies on the development process of the mandibles was inspired by the fact that accessory foramina are present in adult mandibles. The first, and thus far only, research which mentions accessory foramina in fetuses was a study by Chavez-Lomeli et al. (1996) on the development of the human mandibular canal. Authors of that study described the presence of lingual foramina in the majority of the investigated fetal mandibles, and they observed their connections with the alveoli of the mandibular teeth (Chavez-Lomeli et al. 1996). The frequency of occurrence of accessory foramina may be influenced by the developmental modeling processes, starting from the internal surface of the mandible, where the Meckel’s cartilage is found. Accessory foramina present in the midline of the mandible imply their connection to the development of this portion of the mandible, which is formed from the accessory symphyseal ossicles and the medial portions of the opposing Meckel’s cartilages. With the exception of the frequency, which is considerably lower, our study showed no significant differences in the location of the accessory foramina in children and fetuses compared with adult mandibles. Our study confirms previously reported observations (Chavez-Lomeli et al. 1996) that accessory foramina located on the internal surface of the mandible are connected with the mandibular canal. This connection implies that the accessory foramina may be an important route for nerves and vessels.

In conclusion, it can be stated that macroscopic observations revealed the presence of accessory foramina in most of the investigated mandibles, and thus the foramina can be considered as constant anatomical structures and their nomenclature should be unified and standardized.

Ethical approval was obtained for the Department of Anatomy from the Institutional Review Board at Poznan University of Medical Sciences
